# The Comparative Efficacy of Palmitoylethanolamide (PEA) With the Combination of Pregabalin and Nortriptyline on Post-extraction Trigeminal Neuropathy by Using Magnetic Resonance (MR) Neurography: A Randomized Clinical Trial

**DOI:** 10.7759/cureus.54843

**Published:** 2024-02-24

**Authors:** Amlendu Shekhar, Adit Srivastava, Nimisha Verma, Ashish Verma, T P Chaturvedi

**Affiliations:** 1 Department of Oral Medicine and Radiology, Faculty of Dental Sciences, Institute of Medical Sciences, Banaras Hindu University (BHU), Varanasi, IND; 2 Department of Anesthesiology, Institute of Medical Sciences, Banaras Hindu University (BHU), Varanasi, IND; 3 Department of Radiology, Institute of Medical Sciences, Banaras Hindu University (BHU), Varanasi, IND; 4 Department of Orthodontics, Faculty of Dental Sciences, Institute of Medical Sciences, Banaras Hindu University (BHU), Varanasi, IND

**Keywords:** neurosensory testing, orofacial pain, mr neurography, palmitoylethanolamide, pttn

## Abstract

Aim

The aim of this randomized clinical trial is to compare the efficacy of palmitoylethanolamide (PEA) with the combination of pregabalin and nortriptyline in treating post-extraction trigeminal neuropathy using magnetic resonance neurography (MRN).

Methods

The present prospective, randomized controlled trial was conducted on 60 patients (20 in each group). In group I (positive control group), a combination of 75 mg of pregabalin and 10 mg of nortriptyline was administered once daily for the duration of 12 weeks. In group II, 600 mg of palmitoylethanolamide was given twice a day. In group III, a combination therapy of the abovementioned drugs was given. The efficacy of the drug was assessed by measuring pain intensity in terms of the numeric rating scale (NRS) (primary outcome) and changes (signal intensity and nerve thickness) in magnetic resonance neurography (secondary outcome) at various intervals of time. The data was collected and subjected to statistical analysis using the Statistical Package for Social Sciences (SPSS) version 25 (IBM SPSS Statistics, Armonk, NY) at the significance level of P<0.05.

Results

A significant decrease in post-drug mean NRS scores was observed in all three groups. In terms of reduction in the mean NRS, the combination group showed the highest reduction. Palmitoylethanolamide significantly reduces pain scores with negligible side effects.

Conclusion

Palmitoylethanolamide helps in the reduction of mild to moderate pain of painful post-traumatic trigeminal neuropathy (PTTN) with minimal side effects, suggesting that it may be used where the use of the conventional drug is either contraindicated or not feasible.

## Introduction

Iatrogenic painful post-traumatic trigeminal neuropathy (PTTN) caused by tooth extractions results in facial and jaw pain and accounts for 60% of all the nerve injuries in the jaw [1]. Persistent nerve damage leads to disabling neuropathic pain and significant oral dysfunction. Therefore, early diagnosis and timely treatment are critical in improving both patient outcomes and prognosis [2]. To evaluate nerve dysfunction, it is important to use objective testing rather than subjective reporting by the patient to ensure a correct diagnosis [3].

Recent studies have shown the diagnostic reliability of clinical neurosensory testing (NST). However, NST results are not reliable as they cannot pinpoint the exact location of the injury or delineate the anatomy for presurgical planning [4]. As a result, improved diagnostic reliability for painful post-traumatic trigeminal neuropathy is required. Magnetic resonance neurography (MRN) is an imaging technique used to evaluate peripheral nerves and characterize neuropathies. This procedure provides a detailed image of a nerve based on the resonance signal that originates in the nerve itself rather than in the surrounding tissue or fat of the nerve lining. MRN can quantitatively and reliably differentiate normal from injured nerves with high accuracy [5].

The poor outcome of the treatment protocol is a reflection of the limited number of drugs available, their limited efficacy for neuropathic pain, and their side-effect profile, so there is a need for more efficacious and safer drugs for the treatment of neuropathic pain [6]. Studies have shown that palmitoylethanolamide (PEA), an endogenous lipid, is produced in almost every cell by on-demand synthesis when needed and is involved in endogenous protective mechanisms activated as a result of the stimulation of the inflammatory response [7]. There have been multiple prospective observational and randomized PEA trials that have shown PEA to be a good add-on analgesic nutraceutical [8].

The aim of this study was to determine the comparative efficacy of palmitoylethanolamide (PEA) with a combination of pregabalin and nortriptyline in the management of painful post-traumatic trigeminal neuropathy determined using neurosensory testing (NST) and magnetic resonance neurography (MRN).

## Materials and methods

Study design

The present study was a single-center, parallel, randomized controlled trial with a 1:1 allocation ratio. The data was collected from the patients reporting to the tertiary care center in Varanasi, Uttar Pradesh (UP), India, between August 2021 and July 2022. From the Institutional Ethics Committee of of the Institute of Medical Sciences (IMS), Banaras Hindu University, ethical clearance was obtained dated June 23, 2021 (approval number: Dean/2021/EC/2717). The study was registered with the Clinical Trials Registry of India with the number CTRI/2021/08/036091 on August 8, 2021. The study was conducted in accordance with the principles of the Declaration of Helsinki, and written informed consent was obtained from each participant and/or their legal guardian prior to inclusion.

Sample size estimation

In the study by Dessouky et al. [4], the sample size was calculated based on differences in nerve thickness and T2SIR among the case and control groups for inferior alveolar nerve (IAN), lingual nerve (LN), posterior superior alveolar (PSA) nerve, and mental nerve, which were measured using MR neurography. The authors of this study considered an equivalence test for sample size calculations. The sample size was estimated based on the following assumptions: an alpha error of 5% and a study power of 95%. Assuming a 10% dropout rate, the final sample size was calculated to be a minimum of 20 per group.

Study population

Patients over 18 years of age and both genders with complaints of orofacial pain lasting more than three months after extraction were enrolled in the study after fulfilling the inclusion criteria.

Patients were included in the study if they had persistent pain in either the maxillary or mandibular divisions of the trigeminal nerve for more than three months after extraction on the same side of the face. Patients with systemic diseases such as diabetes mellitus, hypertension, hypo- or hyperthyroidism, and renal disease; pregnant or lactating mothers; and patients with symptoms of classic trigeminal neuralgia were excluded. In order to ensure that discomfort reported by the participants was specifically attributable to post-traumatic trigeminal neuropathy (PTTN) and not to other potential differential diagnosis conditions, thorough clinical assessment and diagnostic evaluation were conducted by following the diagnostic criteria given by the International Headache Society (IHS) in its International Classification of Headache Disorder, 3rd Edition (ICHD 3.0) to rule out any alternative diagnosis.

Interventions

**Figure 1:**
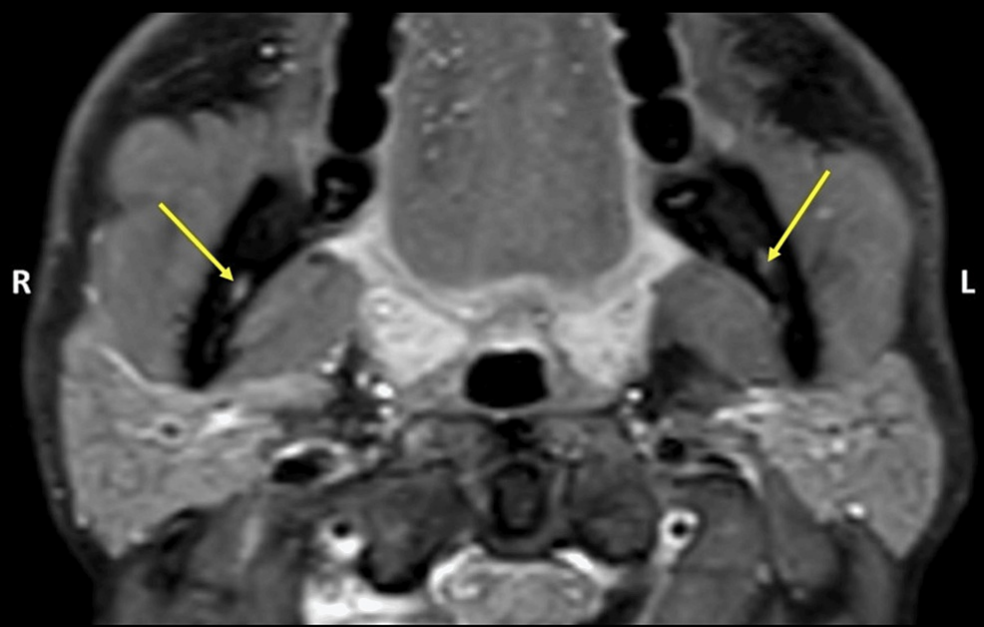
T1-weighted axial section showing the entry of the inferior alveolar nerve in the mandibular canal (yellow arrows)

**Figure 2:**
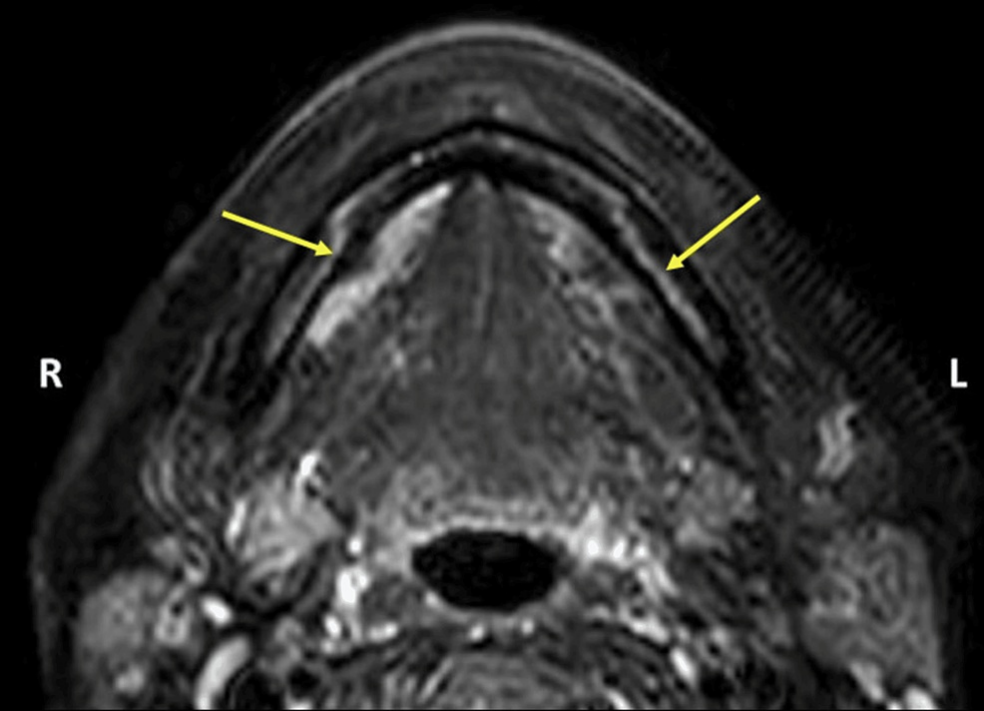
T2-weighted axial section showing the course of the inferior alveolar nerve in the mandible (yellow arrows)

**Figure 3:**
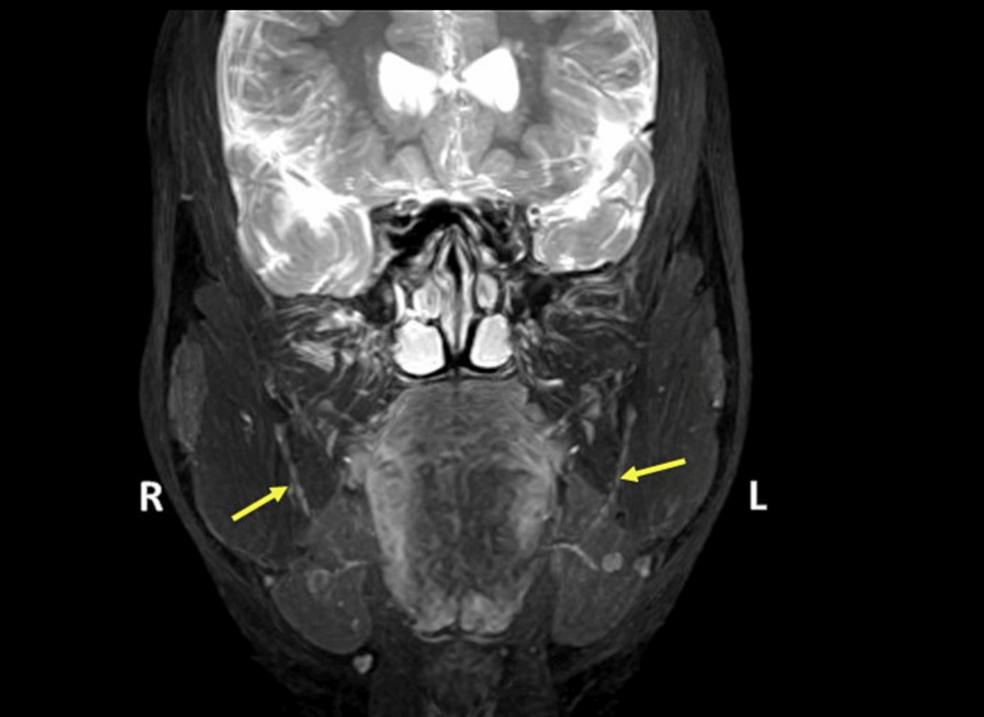
3D coronal PSIF image showing the course of the inferior alveolar nerve (yellow arrows) 3D, three-dimensional; PSIF, posterior spinal instrumentation and fusion

**Figure 4:**
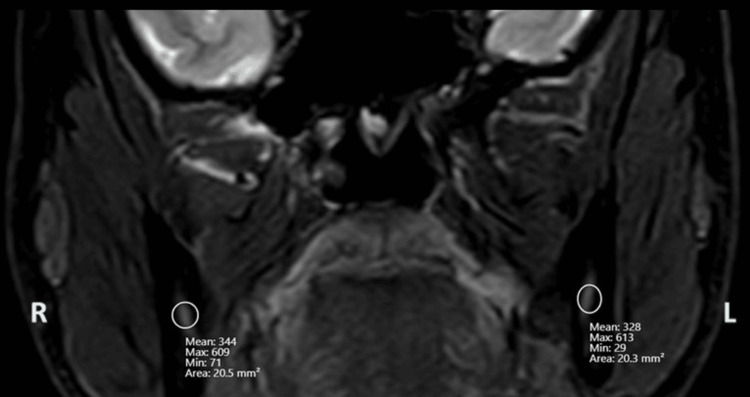
Mean signal intensity measurement for the right and left IAN by drawing the ellipsoidal ROI IAN, inferior alveolar nerve; ROI, region of interest

**Figure 5:**
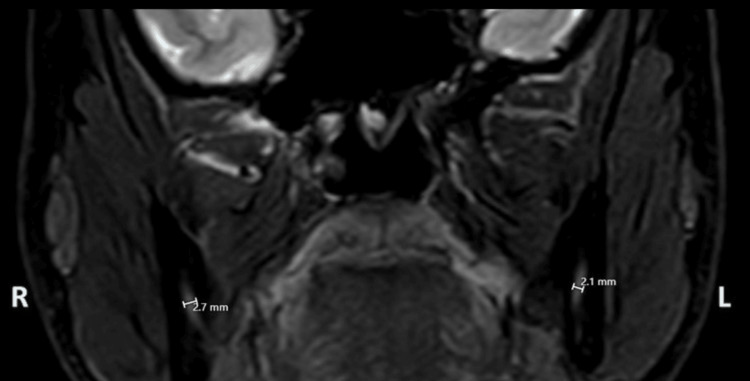
Increased nerve thickness was noted at the right IAN following nerve injury due to extraction IAN: inferior alveolar nerve

Patients were randomly divided into three groups, namely, the active control group (group I), the experimental group (group II), and the combination therapy group (group III). Before the intervention, a pre-treatment magnetic resonance neurography was done at baseline to trace the course of the trigeminal nerve and record signal intensity and nerve thickness (Figures 1-5).

Group I patients received a combination of pregabalin 75 mg and nortriptyline 10 mg once daily during nighttime bedtime for a period of three months. The patients in group II received palmitoylethanolamide (PEA) 600 mg twice daily in the morning and evening for three weeks, followed by once daily in the morning for nine weeks as monotherapy. The patients in group III received a combination therapy (pregabalin 75 mg+nortriptyline 10 mg) once daily during nighttime bedtime and palmitoylethanolamide 600 mg twice daily in the morning and evening for the first three weeks, followed by once daily administration of palmitoylethanolamide in the morning along with pregabalin and nortriptyline for an additional nine weeks. The patients were followed up for a total of three months. After a follow-up period of three months, magnetic resonance neurography was performed at the same site of nerve injury following the same acquisition protocol.

Outcomes

The comparative evaluation of the drug, i.e., PEA, was made on the basis of a reduction in the mean score of pain intensity in terms of numeric rating scale (NRS) (primary outcome) from baseline and after one month and three months and changes (nerve thickness and signal intensity) seen in magnetic resonance neurography from baseline and after three months (secondary outcome).

Randomization

The random allocation sequence was generated using a computer-generated method accessible at www.randomizer.org. Simple randomization was employed as the type of randomization. The implementation process involved Researcher 1 and Researcher 3 enrolling all patients and generating the random allocation sequence. Subsequently, Researcher 2 assigned patients to different groups, and Researcher 4 handled the reporting of magnetic resonance neurography. The study was single-blinded, ensuring that the assignment of patients to specific groups was concealed from the researchers. Statistical analysis was conducted using the Statistical Package for Social Sciences (SPSS) version 25 (IBM SPSS Statistics, Armonk, NY), with data entry and compilation performed in MS Excel (Microsoft Corp., Redmond, WA). The significance level was set at 5%. Normal distribution assessment using the Shapiro-Wilk test indicated that the data followed a normal distribution. Descriptive statistics were employed for demographic data comparison. For intergroup analysis, repeated measures analysis of variance (ANOVA) was applied, followed by post hoc comparisons. Intragroup analysis utilized one-way ANOVA and paired t-test, followed by post hoc comparisons.

## Results

**Figure 6:**
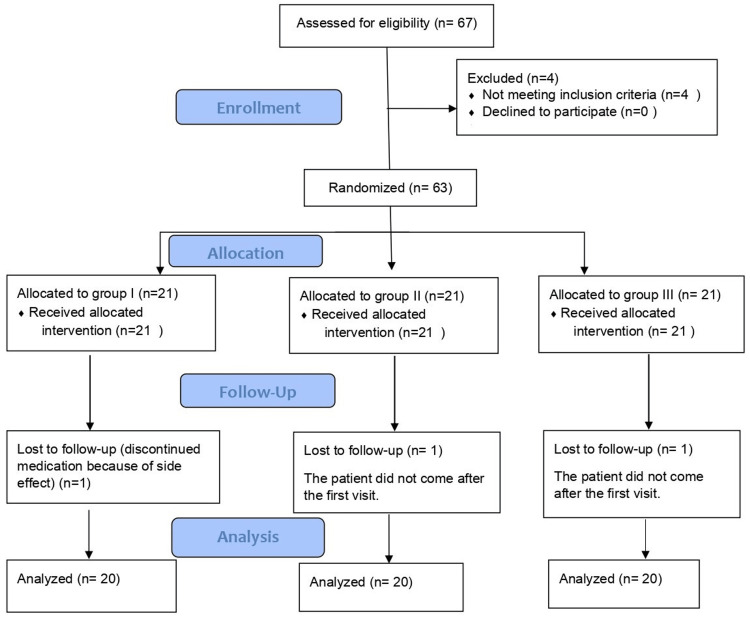
Participant flow diagram (Consolidated Standards of Reporting Trials {CONSORT} 2010)

In this randomized control clinical trial, a total of 67 patients were selected from June 2021 to July 2022 on the basis of inclusion and exclusion criteria, out of which four refused to participate in the study, making a total of 63 patients who were included in the study. The included patients (n=63) were then randomly divided into three groups (n=21 each): group I (positive control group), group II (experimental group), and group III (combination therapy group). During the course of the study, three patients, one from each group, were lost to follow-up; thus, a total of 60 patients (20 in each group) were evaluated (Figure 6).

The demographic characteristics of the study are depicted in Table 1.

**Table 1:** Demographic characteristics of group I, group II, and group III M, male; F, female

Demographic characteristics	Group I (n=20)	Group II (n=20)	Group III (n=20)
Age (in years)	Mean: 45±10	Mean: 43.5±10	Mean: 45±10
Gender	M=3; F=17	M=5; F=15	M=4; F=16
Most commonly extracted tooth	Lower right third molar (n=14)	Lower right third molar (n=15)	Lower right third molar (n=15)
Most common presenting clinical symptoms along with pain	Heaviness and feeling of swelling	Heaviness and feeling of swelling	Heaviness and feeling of swelling

Quantitative neurosensory testing was done by a single examiner. The five scores of sensory impairment were given, which denoted normal, mild, moderate, severe, and complete loss. Most of the patients (n=55) fell into mild to moderate cases, and only two patients showed a complete loss. Most of the patients (n=51) showed reduced tactile response for 0.07 g of force by Semmes-Weinstein monofilament, and only two patients showed reduced tactile response for even 300 g of force.

Primary outcome

The primary outcome was assessed using a clinical numeric rating scale (NRS). The mean NRS of group I at baseline was 5.40±1.14, group II was 5.60±1.14, and group III was 5.30±1.08, so there was no significant difference observed when NRS was measured at baseline in the three groups (P>0.05) After one month of follow-up, there was a significant decrease in the mean pain NRS in all three groups (P<0.05). In intergroup comparison, there was a significant decrease in post-drug mean NRS scores in all three groups from baseline to three-month follow-up (P<0.05). The greatest reduction in the mean NRS was observed in the combination therapy group, i.e., from the mean of 5.30 to the mean of 1.50 (Table 2).

**Table 2:** Intergroup comparison of the numeric rating scale (NRS), nerve signal intensity, and nerve thickness in different groups The total range of the NRS scale=0-10. Intergroup analysis using repeated measures ANOVA test *Insignificant **Significant ANOVA, analysis of variance; au, arbitrary unit

Parameters	Groups	Mean	Standard deviation	P-value
NRS at baseline	Group I	5.40	1.14	0.692*
	Group II	5.60	1.14	
	Group III	5.30	1.08	
NRS after one month	Group I	3.72	1.16	0.001**
	Group II	4.07	1.17	
	Group III	2.80	0.76	
NRS after three months	Group I	1.90	0.79	0.001**
	Group II	2.35	0.69	
	Group III	1.50	0.51	
Signal intensity at baseline (au)	Group I	3.60	0.40	0.408*
	Group II	3.46	0.60	
	Group III	3.35	0.70	
Signal intensity after three months (au)	Group I	2.32	0.29	0.000**
	Group II	2.16	0.47	
	Group III	1.66	0.55	
Nerve thickness at baseline (mm^2^)	Group I	3.25	0.71	0.630*
	Group II	3.06	0.80	
	Group III	3.26	0.69	
Nerve thickness after three months (mm^2^)	Group I	2.91	0.70	0.351*
	Group II	2.72	0.76	
	Group III	2.58	0.66	

A post hoc analysis was done for intergroup comparison, and there was no significant difference found when NRS was measured at baseline, but after one month of follow-up, only group III showed a significant difference in the mean NRS when compared to group II. After three months of follow-up, there was a significant difference in the mean NRS between group II and group III, and group III showed better results than group II. Although after three months there was a reduction in the mean NRS in groups I and II, the difference between them was not significant, and group I showed better results than group II. The intragroup comparison of the mean NRS showed a significant difference in values when measured at baseline, after one month, and after three months. So, as far as pain is concerned, palmitoylethanolamide as a sole therapy showed a significant reduction in pain intensity at each consecutive follow-up period (Table 3).

**Table 3:** Intragroup comparison of the mean NRS for different groups All the groups showed significant results when the mean NRS was compared from baseline, after one month, and after three months (P<0.05) *Significant NRS: numeric rating scale

Group		Mean difference	P-value
Positive control group	NRS at baseline versus NRS after one month	1.675^*^	0.000
	NRS at baseline versus NRS after three months	3.500^*^	0.000
	NRS after one month versus NRS after three months	1.825^*^	0.000
Experimental group	NRS at baseline versus NRS after one month	1.525^*^	0.000
	NRS at baseline versus NRS after three months	3.250^*^	0.000
	NRS after one month versus NRS after three months	1.725^*^	0.000
Combination therapy group	NRS at baseline versus NRS after one month	2.500^*^	0.000
	NRS at baseline versus NRS after three months	3.800^*^	0.000
	NRS after one month versus NRS after three months	1.300^*^	0.000

Secondary outcome

Pre- and post-treatment MR neurography was done for all the patients. Nerve injuries were classified on the basis of the Sunderland classification of nerve injuries (Table 4).

**Table 4:** The nerve injury on MR neurography was classified according to the Sunderland classification of nerve injuries [4] MR: magnetic resonance

Class of injury	Features of MR neurography
I	Qualitative, homogeneous increased T2 signal of the nerve with no change in caliber; quantitative, no changes
II	Qualitative, homogeneous increased T2 signal of the nerve and mild nerve thickening and perineural fibrosis; quantitative, <50% larger than contralateral/normal nerve
III	Qualitative, homogeneous increased T2 signal of the nerve and moderate to marked nerve thickening and perineural fibrosis; quantitative, >50% larger than contralateral/normal nerve
IV	Qualitative, heterogeneous increased T2 signal of the nerve and focal enlargement in the otherwise continuous nerve (neuroma in continuity) and perineural and intraneural fibrosis; quantitative, focal swelling with heterogeneous T2 signal or fascicular disruption
V	Qualitative, discontinuous nerve with end-bulb neuroma; quantitative, complete disruption with gap and end-bulb neuroma

On the basis of that, the type of nerve injury causing neuropathy was established. Most of the patients (n=51) had type II nerve injury, very few of them (n=7) had type III, and only two patients had type V nerve injury. The most common nerve involved was the inferior alveolar nerve (n=45), followed by the mental nerve (n=11), the lingual nerve (n=10), and the posterior superior alveolar nerve (n=2). All the patients showed increased T2 signal intensity on the affected side of the nerve when compared to the normal side with the inferior alveolar nerve, showing the greatest change in nerve signal intensity.

The comparison was done for the change in signal intensity and the thickness of the nerve at baseline and after three months. In intergroup comparison for the change in signal intensity, there was a significant reduction in the mean signal intensity after three months of follow-up in all three groups (P<0.05), and group III showed the greatest reduction in signal intensity (Table 2).

In post hoc analysis for the change in signal intensity, there was no significant difference found in the mean signal intensity at baseline among the three groups (P>0.05). After three months, the difference in the change in signal intensity between group I and group II was not significant, and group II showed slightly better results than group I, and group III showed the best result when compared to group I and group II in terms of reduction in signal intensity post treatment. In intergroup analysis, the comparison between nerve thickness at baseline and after three months was done between groups, and we found no significant change in nerve thickness at baseline and after three months among groups (P>0.05) (Table 2).

In post hoc analysis, comparisons between groups were made on the basis of the change in nerve thickness post treatment, and we found no significant difference at baseline and after three months. Group III showed better results than group II and group I, and group II showed slightly better results than group I. Intragroup analysis was done to compare the changes seen in signal intensity and nerve thickness at baseline and after three months of follow-up. We found that there was a significant reduction in signal intensity and nerve thickness in all the groups after three months (Table 5).

**Table 5:** Intragroup comparison of signal intensity and nerve thickness All the groups have shown significant results for changes seen in nerve signal intensity and nerve thickness at different time intervals (P<0.05) au: arbitrary unit

Group			Mean	Standard deviation	P-value
Positive control group	Signal intensity (au)	Baseline	3.600	0.4078	0.000
		After three months	2.325	0.2900	
	Nerve thickness (mm^2^)	Baseline	3.255	0.7134	0.000
		After three months	2.910	0.7085	
Experimental group	Signal intensity (au)	Baseline	3.460	0.6091	0.000
		After three months	2.160	0.4773	
	Nerve thickness (mm^2^)	Baseline	3.065	0.8008	0.000
		After three months	2.725	0.7691	
Combination therapy group	Signal intensity (au)	Baseline	3.350	0.7052	0.000
		After three months	1.660	0.5586	
	Nerve thickness (mm^2^)	Baseline	3.265	0.6961	0.000
		After three months	2.580	0.6685	

Adverse reactions to drugs

All the patients in group I showed side effects, the most common being dizziness, GI disturbance, and drowsiness; two patients showed palpitations. However, with time, the intensity of these side effects decreased. Only one patient in group II showed GI disturbance (acid reflux), and the rest of the patients tolerated well palmitoylethanolamide. The patients of group III showed drowsiness, swollen body, and gastric upset, and that too resolved after a few weeks of treatment.

## Discussion

Our study found that patients with painful post-traumatic trigeminal neuropathy had a mean age of 45±10 years, with a female predominance (male/female ratio: 1:4). Similar studies by Peñarrocha et al. [9], Dessouky et al. [4], Zuniga et al. [10], and Gregg [11] also reported female predominance in their respective studies of trigeminal neuropathy. Bouhassira et al. [12] found a higher prevalence of chronic pain with neuropathic characteristics in females, which could be attributed to nutritional deficiencies or hormonal changes. Singh and Lone [13] and Benoliel et al. [14] reported a male preponderance in their studies on post-traumatic sensory disturbance in mandibular fractures, which may be attributed to the higher prevalence of mandibular fractures in males.

Regarding neurosensory deficits, our study found that 32% of the patients had a feeling of heaviness, 26% had a burning sensation, and 21% had a crawling and tingling sensation. Gregg [11] found that the patients most often described their pain as numbing, pulling, itching, crawling, annoying, and heavy. Renton et al. [15] found that numbness was the predominant accompanying symptom in patients with neurosensory dysfunction.

Several studies examined various symptoms of neurosensory dysfunction in patients with post-traumatic trigeminal neuropathy (PTTN). The most commonly reported symptoms were numbness, allodynia, hyperalgesia, burning, tingling, floppy, and crawling. Numbness was the predominant symptom [10,11,15]. Quantitative neurosensory testing (NST) is commonly used to diagnose PTTN, but its reliability is limited in the first three months after injury. In addition, NST cannot determine the exact location of injury or anatomy for preoperative planning, and it has lower positive and negative predictive values for inferior alveolar nerve (IAN) injuries [4,10]. MR neurography is a noninvasive diagnostic tool that can accurately depict trigeminal nerve anatomy in multiple orthogonal planes and distinguish between normal and abnormal peripheral trigeminal nerves [5]. MR neurography can also identify imaging alterations in nerve caliber and T2 signal intensity, which contributes to a more accurate diagnosis of neuropathy. MR neurography is more reliable and objective than NST and can significantly impact clinical management. Routine imaging techniques used for dentoalveolar injuries, such as Intra-oral periapical radiographs (IOPAs), orthopantomograph (OPG), and cone-beam computed tomography (CBCT), do not provide additional information in PTTN cases. The inferior alveolar nerve was most commonly affected in PTTN in our case, followed by the mental nerve, lingual nerve, and posterior superior alveolar nerve. Damage to the inferior alveolar nerve is due to its close proximity to the apical portion of the third molar, where the nerve is located [16].

Several studies have investigated nerve injury after the extraction of the mandibular third molar. Zuniga et al. [10] reported that the inferior alveolar nerve was injured in 40 patients, whereas the lingual nerve was involved in 20 patients. In contrast, studies by Dessouky et al. [4]. and Renton et al. [15] found that the lingual nerve was most commonly injured after molar extraction, followed by the inferior alveolar nerve. In general, the surgical removal of mandibular third molars is a common procedure worldwide, often involving branches of the mandibular nerve, including the mental nerve [17]. Consequently, cases involving the posterior superior alveolar nerve are rare.

Regarding treatment, no study has compared MR neurography parameters before and after treatment, especially nerve thickness and signal intensity. Our study is the first to do so. Most patients in our study had mild to moderate nerve injury according to the Sunderland classification, with only two patients suffering complete nerve loss. In contrast, studies by Zuniga et al. [10] and Dessouky et al. [4]. found that severe cases predominated, with few cases showing complete nerve loss.

Gabapentinoids such as pregabalin are the first choice in the treatment of neuropathic pain because they act supra-spinally to stimulate descending inhibition and reduce antihypersensitivity in peripheral nerve injury [18]. Nortriptyline, on the other hand, increases the availability of norepinephrine by inhibiting neuronal uptake, potentiating the activity of descending inhibitory pathways, and relieving pain [18]. However, clinical trials have reported the limited efficacy of pregabalin as a single therapy [19]. In our study, the patients in group I and group III were administered 10 mg of nortriptyline daily in combination with 75 mg of pregabalin for 12 weeks.

Based on NRS scores at pain screening, we classified pain intensity as mild, moderate, or severe. Most of our patients had moderate pain, consistent with the MR neurography finding of moderate nerve involvement (Sunderland classification grade II) [20]. Our treatment protocol proved effective for mild to moderate pain.

Palmitoylethanolamide (PEA) is an endogenous lipid mediator known for its neuroprotective, anti-inflammatory, and analgesic functions. It is found in soy lecithin, egg yolk, and peanut flour. PEA is an endogenous fatty acid amide that exerts its therapeutic effects through various mechanisms, primarily involving the modulation of the endocannabinoid system and anti-inflammatory pathways. During its endocannabinoid system modulation, PEA acts as an endogenous agonist of the peroxisome proliferator-activated receptor alpha (PPAR-α), a nuclear receptor involved in regulating inflammation and pain. The activation of PPAR-α by PEA leads to the downstream modulation of gene expression, resulting in anti-inflammatory and analgesic effects. By its anti-inflammatory effect, PEA suppresses the release of pro-inflammatory mediators such as tumor necrosis factor-alpha (TNF-α), interleukin 1 beta (IL-1β), and prostaglandins. By inhibiting the activation of mast cells and microglia, PEA helps to attenuate neuroinflammation, thereby reducing pain and neuropathic symptoms. In neuroprotection, PEA exhibits neuroprotective properties by promoting neuronal survival and inhibiting neurodegenerative processes. It modulates neurotrophic factors such as brain-derived neurotrophic factor (BDNF), which plays crucial roles in neuronal repair and plasticity. By the modulation of ion channels, PEA interacts with transient receptor potential vanilloid type 1 (TRPV1) channels, leading to the modulation of nociceptive signaling and pain perception. By desensitizing TRPV1 channels, PEA can attenuate pain transmission and alleviate neuropathic pain. Fotio et al. [21] reported a significant reduction in neuropathic pain with the use of palmitoylethanolamide, indicating its analgesic, anti-inflammatory, and neuroprotective properties.

Chronic pain is a common condition that significantly affects the patients' quality of life. Unfortunately, many existing therapies do not provide adequate relief and are often associated with serious side effects, especially in elderly and frail patients. One potential solution is palmitoylethanolamide (PEA), which has been shown to reduce the need for rescue medications and minimize the dosage of painkillers, thereby reducing their side effects [22-24]. In clinical trials, PEA as monotherapy has been shown to be effective in relieving pain in a number of chronic pain conditions, including postherpetic neuralgia, diabetic neuropathy, and chemotherapy-induced neuropathy [8,25]. A study by Ottaviani et al. [26] in 2019 reported a significant difference in pain reduction between PEA and placebo in the patients with burning mouth syndrome. Furthermore, a study by Steels et al. [27] in 2019 also reported significant pain reduction by PEA when compared to placebo. Similarly, a study by Pickering et al. [28] in 2022 also reported significant improvement in the neuropathic pain scale in patients with diabetic neuropathy. A meta-analysis conducted by Lang-Illievich et al. [29] in 2023 indicates a consistently favorable effect of palmitoylethanolamide (PEA) compared to placebo or active comparators in the management of chronic pain. Across multiple studies, PEA demonstrated an improvement in functional status and quality of life among patients. Furthermore, the reported side effects associated with PEA were minimal and of negligible clinical significance. However, these findings are not in concordance with our study as we did not find PEA sole therapy to be superior in the reduction of pain or MR neurography-based parameters. However, the intragroup comparison of baseline and three-month follow-up of PEA revealed a significant reduction in pain scores and MR neurography parameters, suggesting that it may be used where the use of the conventional drug is either contraindicated or not feasible.

While further studies with larger samples and longer follow-up periods are needed to fully understand the benefits and limitations of PEA, it can show promising results as a safe and effective alternative for patients who cannot tolerate or benefit from conventional therapies.

None of the clinical trials compared the efficacy of palmitoylethanolamide with a combination of pregabalin and nortriptyline in patients with painful post-traumatic trigeminal neuropathy. This study is the first of its kind to demonstrate differences in MR neurography parameters before and after treatment. Although this study had some limitations, such as a small sample size and a short follow-up period, it provides evidence that PEA can provide significant improvement in pain and other symptoms of painful post-traumatic trigeminal neuropathy with minimal side effects. For future studies, the researcher suggests larger sample sizes, longer follow-up periods, and the consideration of additional MR neurography parameters such as apparent diffusion coefficient (ADC) and fractional anisotropy (FA).

## Conclusions

In conclusion, palmitoylethanolamide may be a valuable addition to routine clinical practice in the treatment of chronic pain conditions. However, further studies are needed to confirm its efficacy and better understand its limitations. By continuing to research and develop new therapies, clinicians and diagnosticians can improve the quality of life for patients suffering from neuropathic pain.
